# 2512. A Decade in Review: An Analysis of the Qualified Infectious Disease Product (QIDP) Designation

**DOI:** 10.1093/ofid/ofad500.2130

**Published:** 2023-11-27

**Authors:** Cem Atillasoy, Lina Elmansy, Panagiotis Gourlias

**Affiliations:** Yale School Of Medicine, new haven, Connecticut; Yale School Of Medicine, new haven, Connecticut; University of Pittsburgh School of Medicine, Pittsburgh, Pennsylvania

## Abstract

**Background:**

The development of new antibiotics is a critical public health need, as antibiotic-resistant infections are responsible for tens of thousands of deaths each year in the U.S. In response, the QIDP (Qualified Infectious Disease Product) designation was established by the FDA under the Generating Antibiotic Incentives Now (GAIN) Act in 2012. The program incentivizes the development of new drugs to treat serious or life-threatening infections caused by bacteria or fungi that are resistant to existing treatments. These incentives include priority review and 5 additional years of market exclusivity for drugs that receive QIDP designation and are subsequently approved by the FDA.

**Methods:**

We analyzed the FDA Database entitled, “Compilation of CDER New Molecular Entity (NME) Drug and New Biologic Approvals” for antimicrobial therapies approved after 2012. Antimicrobial therapies were defined as antibiotics and antifungals, excluding antivirals and antiparasitics which are ineligible for QIDP. We compared the status of the QIDP designation in drugs that gained FDA approval over two 5-year periods (2013-2017 vs. 2018-2022). We also analyzed the types of infections and pathogens targeted by these drugs.

**Results:**

Since the GAIN Act in 2012, there have been 25 antimicrobial therapies approved, 20 with the QIDP designation (Table 1). 9 drugs with QIDP status were approved (out of 13 antimicrobials) from 2013-2017; whereas 2018-2022 had 11 drugs approved with QIDP status (out of 12 antimicrobials) (Figure 1). There was no significant difference in QIDP approvals over the two periods (69% vs. 92%, p=0.32).

Of the 20 drugs approved with the QIDP designation, 9 are indicated to treat genitourinary infections, 6 to treat gastrointestinal infections, 5 to treat skin infections, 3 to treat pulmonary infections and only 1 to treat systemic infections. Furthermore, 15 of the drugs targeted bacterial pathogens, 4 targeted fungal pathogens and 1 targeted mycobacteria (Table 2).

**Figure:**
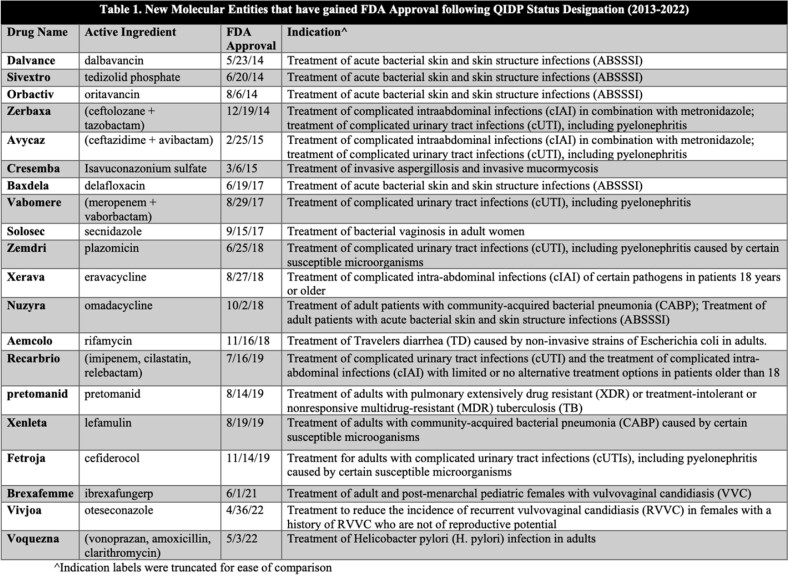

**Figure:**
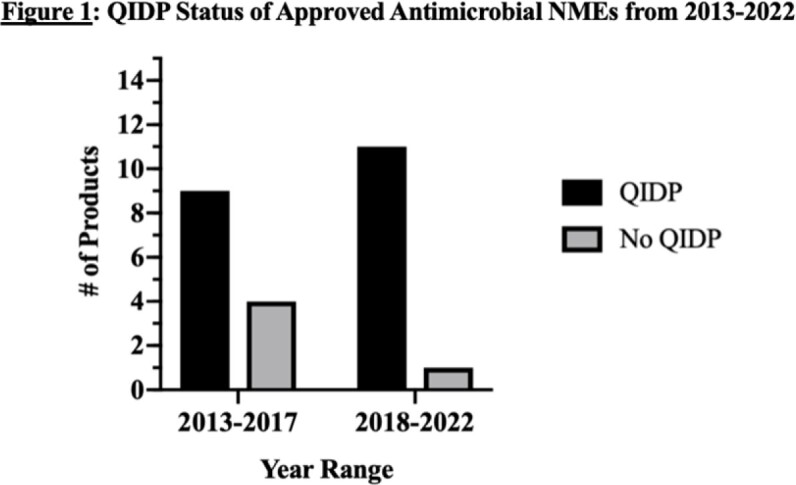

**Figure:**
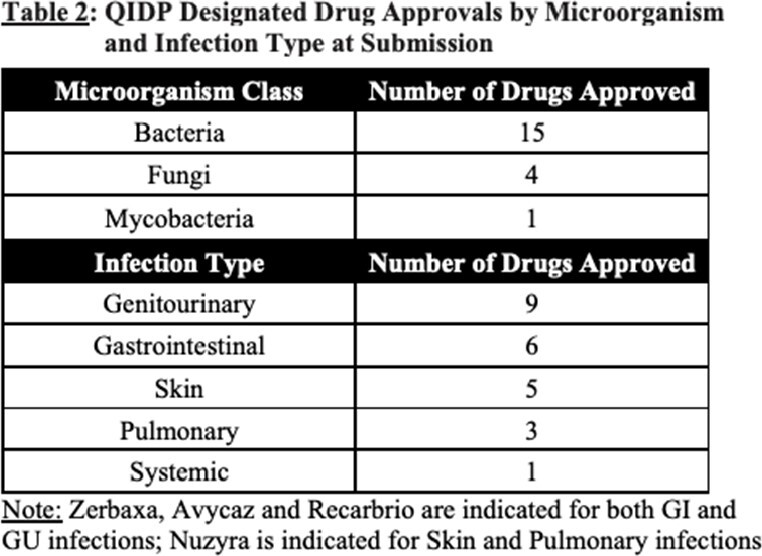

**Conclusion:**

Our findings indicate that the QIDP designation has been moderately effective in generating renewed interest in the development of antibiotics. Additional strategies are needed to encourage continued infectious disease R&D efforts, especially around areas of unmet need such as systemic infection and fungal pathogens.

**Disclosures:**

**All Authors**: No reported disclosures

